# Controlled p-Type Doping of MoS_2_ Monolayer by Copper Chloride

**DOI:** 10.3390/nano12172893

**Published:** 2022-08-23

**Authors:** Sangyeon Pak

**Affiliations:** School of Electronic and Electrical Engineering, Hongik University, Seoul 04066, Korea; spak@hongik.ac.kr

**Keywords:** MoS_2_ monolayer, copper chloride, transition metal chloride, p-type doping, spin coating

## Abstract

Electronic devices based on two-dimensional (2D) MoS_2_ show great promise as future building blocks in electronic circuits due to their outstanding electrical, optical, and mechanical properties. Despite the high importance of doping of these 2D materials for designing field-effect transistors (FETs) and logic circuits, a simple and controllable doping methodology still needs to be developed in order to tailor their device properties. Here, we found a simple and effective chemical doping strategy for MoS_2_ monolayers using CuCl_2_ solution. The CuCl_2_ solution was simply spin-coated on MoS_2_ with different concentrations under ambient conditions for effectively p-doping the MoS_2_ monolayers. This was systematically analyzed using various spectroscopic measurements using Raman, photoluminescence, and X-ray photoelectron and electrical measurements by observing the change in transfer and output characteristics of MoS_2_ FETs before and after CuCl_2_ doping, showing effective p-type doping behaviors as observed through the shift of threshold voltages (Vth) and reducing the ON and OFF current level. Our results open the possibility of providing effective and simple doping strategies for 2D materials and other nanomaterials without causing any detrimental damage.

## 1. Introduction

Monolayer transition metal dichalcogenides (TMDCs) have been considered to be the next-generation semiconducting channel materials because of their incredible electronic and mechanical properties that make them suitable for flexible, wearable, and transparent devices [1,2,3,4,5]. In addition, their ideally dangling bond-free surface and atomic thickness show promise for van der Waals integration on various substrates/materials and in reducing short channel effect, thus becoming candidates for the semiconducting channel materials in nano-scaled electronics and optoelectronics devices [2,6,7,8]. Especially, the field-effect transistors (FETs) composed of TMDC monolayers show high carrier mobility, large On/Off ratio (>10^8^), and low power consumption, which have inspired experimental research in advancing FET performance of these devices [5,9,10,11,12,13,14].

To implement the TMDC monolayers for practical electronic device applications, the device properties need to be tailored to show the desired output characteristics of electronic devices. One way to achieve the desired device characteristics is through doping [3,15,16,17,18,19]. The doping of 2D materials is recognized to be the key to precisely controlling their fundamental properties, based on the history of the contemporary Si or III–V-based semiconductors. Ion implantation is one of the possible doping techniques. However, this uses high energy and can be detrimental to atomically thin 2D crystals. On the other hand, chemical doping is potentially more advantageous compared to the ion implantation method, as the chemical doping is generally based on a charge transfer by chemical potential of adsorbed organic molecules and leads to less damage in 2D crystal structures [18,20,21,22,23]. The chemical doping of 2D TMDCs have been mostly relied on employing self-assemble monolayer (SAM) techniques [3,9], substitutional doping [17,24], and passivation of sulfur vacancy defects [5,25,26]. However, using such techniques, it is difficult to tune the amount of doping, and they generally require a controlled doping environment. Therefore, it is required to find a convenient method to modulate electrical/optical properties, as well as the electronic device properties.

Herein, we report a simple and controllable doping method for 2D MoS_2_ using copper (II) chloride (CuCl_2_) performed at ambient conditions. In this process, the CuCl_2_ is dissolved in ethanol, and the solution is simply spin-coated onto 2D MoS_2_ to effectively modulate charge carrier densities without any damage to MoS_2_ crystals and their devices. The change in doping was analytically confirmed through Raman, photoluminescence (PL), and X-ray photoelectron spectroscopy (XPS), showing the p-type doping effect on 2D MoS_2_. We further confirmed the feasibility of this doping process by simply coating the different concentrations of CuCl_2_ solution onto the back-gated MoS_2_ transistors, showing effective p-type doping behaviors as observed through the shift of threshold voltages (Vth) and reducing the ON and OFF current levels. These findings pave an important pathway toward modulating 2D materials and devices and designing logic devices based on 2D materials.

## 2. Materials and Methods

Synthesis of monolayer MoS_2_: Monolayer MoS_2_ was synthesized on a SiO_2_ (300 nm)/Si substrate using the previously reported thermal chemical vapor deposition (CVD) method [27,28]. Here, 0.05 mg of MoO_3_ precursors were prepared by dissolving the MoO_3_ powders into ammonium hydroxide (NH_4_OH) solution and loading the MoO_3_ onto alumina boats using a micropipette. The SiO_2_/Si substrate was placed above the alumina boat with the substrate placed faced down. The growth was carried in a 2-inch quartz tube, an alumina boat containing 100 mg of sulfur powder was placed upstream, while the alumina boat containing 0.05 mg of MoO_3,_ and substrate was placed downstream in the middle of the CVD furnace. The growth was carried out at 750 °C for 10 min, and the furnace was naturally cooled down to room temperature. MoS_2_ crystal sizes around 30–50 μm were obtained from the CVD synthesis.

Fabrication and measurement of MoS_2_ transistors: The synthesized MoS_2_ monolayers were transferred onto HfO_2_/Si substrate using polystyrene (PS) film as the transferring medium. The PS film (M_W_~192,000) was spin-coated onto MoS_2_/SiO_2_/Si substrate and the film was detached during the transfer process while the film was floated on DI water. The detached PS film with MoS_2_ was dried in air for 2 h and transferred onto HfO_2_/Si substrate for device fabrication. The source and drain electrode pads were patterned using photolithography, and 5 nm Ti/40 nm Au electrodes were deposited using a thermal evaporator. The devices were annealed at 150 °C for 1 h under vacuum conditions. The electrical properties were measured using semiconductor parameter analyzer (Keithley 4200A-SCS) and MS Tech probe station.

Characterization of MoS_2_: The Raman and PL measurements were carried out using Alpha 300 R confocal Raman spectroscopy with 532 nm laser. AFM measurement was carried out using XE7 (Park Systems, Suwon, Korea). XPS measurement was performed using NEXSA (Thermofisher Scientific, Waltham, MA, USA).

## 3. Results

**Figure 1:**
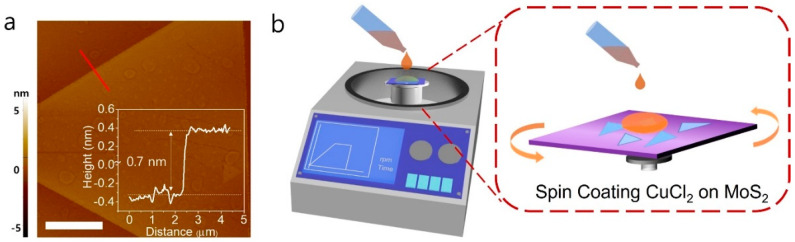
CuCl_2_ doping process for MoS_2_ monolayers. (**a**) AFM topography image of MoS_2_ monolayers showing its atomic thickness around 0.7 nm. Scale bar: 5 μm. (**b**) Schematic illustration of CuCl_2_ doping process. The as-synthesized MoS_2_ monolayers on SiO_2_/Si substrate were placed on a spin coater, and CuCl_2_ solution was dropped onto the substrate for spin coating at 3000 RPM.

MoS_2_ monolayers were synthesized on a SiO_2_ (300 nm)/Si substrate using a chemical vapor deposition (CVD). Figure 1a shows the CVD-grown monolayered MoS_2_ profiled by atomic force microscopy (AFM) height measurement. The morphology and thickness of the as-grown MoS_2_ show its thickness around 0.7 nm, confirming the single-layered thickness. For doping of MoS_2_, CuCl_2_ was employed in this study as the metal chlorides offer a wide range of doping molecules, have been frequently employed to modulate electrical properties of graphene, and are known to be strong electron acceptors [29,30,31]. The metal chloride generally acts as a strong electron acceptor due to the high electronegativity of chlorine compared to molybdenum or sulfur [31]. It should be noted that CuCl_2_ has never been used for doping 2D MoS_2_. Figure 1b illustrates our simple CuCl_2_ doping process. CuCl_2_ was firstly dissolved in ethanol at different molar concentrations (0.5 M and 1 M). The as-grown MoS_2_ layer on SiO_2_ was then placed on a spin coater, and CuCl_2_ solution was dropped onto the as-grown MoS_2,_ followed by the spin coating at 3000 RPM. The doped MoS_2_ samples were then dried on a hot plate at a mild temperature below 90 °C. All of the doping processes were performed in ambient conditions.

**Figure 2:**
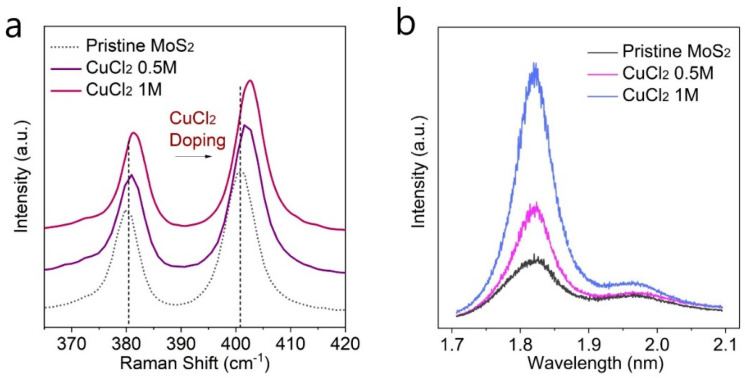
(**a**) Raman and (**b**) PL measurements before and after CuCl_2_ doping. 0.5 M and 1 M CuCl_2_ solutions were employed for doping. After CuCl_2_ doping, the Raman spectrum of MoS_2_ was blue-shifted and PL intensity was largely increased, showing p-type doping effect on MoS_2_.

In order to understand the effect of CuCl_2_ doping on MoS_2_ monolayers, we first performed Raman and PL analysis of the pristine MoS_2_ and CuCl_2_-doped MoS_2_ as shown in Figure 2a,b. Figure 2a shows the Raman spectrum of pristine MoS_2_ (dotted grey line), 0.5 M CuCl_2_-doped MoS_2_ (purple line), and 1 M CuCl_2_-doped MoS_2_ (magenta). The Raman spectrum of pristine MoS_2_ shows two characteristic peaks located at around 381 cm^−1^ and 400 cm^−1^, which correspond to the in-plane E^1^_2g_ and out-of-plane A_1g_ vibrational modes, respectively. As the CuCl_2_ is doped onto MoS_2_, the Raman peaks of MoS_2_ were monotonically blue shifted with increasing the CuCl_2_ doping concentrations. Such a trend of shifting in Raman peaks is a clear signature that carrier concentrations were changed without damaging the crystal structure, and can be understood as a CuCl_2_-induced p-type doping effect, which is in agreement with the previous studies [9,32]. Figure 2b shows PL spectra measured for the pristine MoS_2_ and CuCl_2_-doped MoS_2_. The pristine MoS_2_ shows direct bandgap PL emission at around 1.82 eV. As the MoS_2_ monolayers were doped with CuCl_2_, the PL intensity was largely increased. It has been widely accepted that the PL intensity of 2D MoS_2_ is strongly affected by carrier concentrations. The increased PL intensity of MoS_2_ monolayer can be due to the reduced trion formation and strongly increased exciton radiative recombination rates through decreasing the carrier concentrations of CuCl_2_-doped MoS_2_ monolayers [33]. Therefore, CuCl_2_ doping of MoS_2_ monolayer decreases carrier concentrations due to the strong electron accepting nature of CuCl_2_, which was observed through Raman and PL measurements.

**Figure 3:**
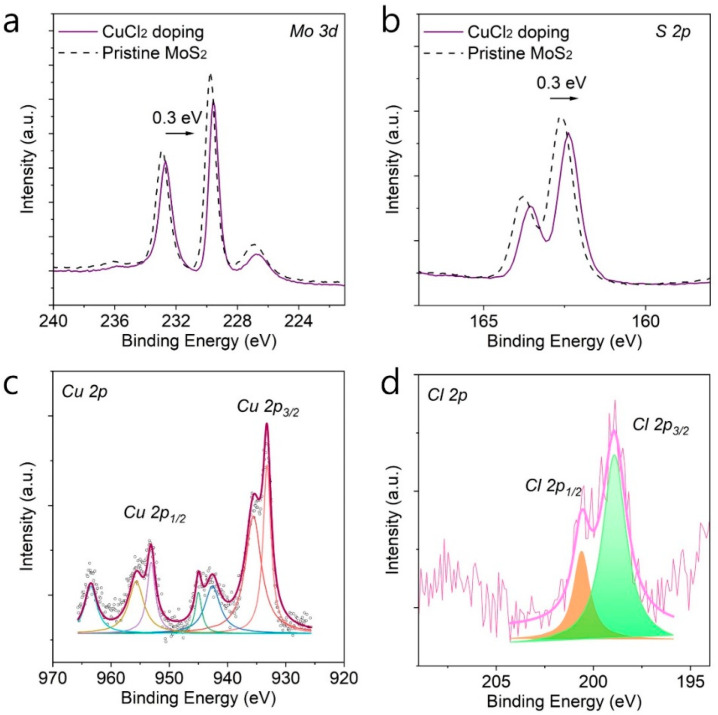
X-ray photoelectron spectroscopy (XPS) measurements before and after CuCl_2_ doping. XPS spectrum of (**a**) Mo 3d and (**b**) S 2p before and after CuCl_2_ doping. The shift of the binding energies to lower energy indicates lowered Fermi level in MoS_2_. XPS spectrum of (**c**) Cu 2p and (**d**) Cl 2p was found in CuCl_2_ doped MoS_2_ film, demonstrating the CuCl_2_ is doped onto MoS_2_.

To further confirm the effect of CuCl_2_ doping and the chemical state of CuCl_2_ molecules, we performed X-ray photoelectron (XPS) analysis as shown in Figure 3a–d. XPS analysis was performed for both pristine MoS_2_ and CuCl_2_-doped MoS_2_, and we compared any change in the binding energies. Figure 3a,b show the main binding energy of MoS_2_, Mo 3d and S 2p peaks, and we compared the change of binding energies before and after CuCl_2_ doping. It is clearly observable that the binding energies of both Mo 3d and S 2p peaks shifted toward lower binding energy by about 0.3 eV. The shift to a lower binding energy in semiconducting MoS_2_ can be attributed to the shift of Fermi-level energy toward the valence band, which results in the change of binding energies in MoS_2_, and the results agree with the Raman and PL analysis that show the p-type doping effect of CuCl_2_ on MoS_2_.

XPS analysis on CuCl_2_-doped MoS_2_ also showed the chemical state of CuCl_2_ as shown in Figure 3c,d. Figure 3c and d show the high-resolution XPS peaks of Cu 2p and Cl 2p peaks, respectively, which were recorded from the CuCl_2_-doped MoS_2_ sample. As shown in Figure 3c, it can be seen that the binding energies of Cu 2p are composed of the main characteristic doublet peaks centered at around 953 eV and 933.3 eV, which correspond to Cu 2p_1/2_ and Cu 2p_3/2_, respectively, and other satellite peaks [34]. The difference between the two peaks is around 19 eV, which is in good agreement with the value reported in the literature [35]. Cl 2p peaks can be deconvoluted into two main doublets, which are found at 200.6 eV and 198.9 eV and correspond to Cl 2p_1/2_ and Cl 2p_3/2_, respectively. The peak difference of the two peaks is around 1.7 eV, which is in good agreement with the value reported in the literature [36]. The XPS results and the presence of Cu 2p and Cl 2p binding energies confirm the presence of CuCl_2_ and the effective p-type doping on MoS_2_.

**Figure 4:**
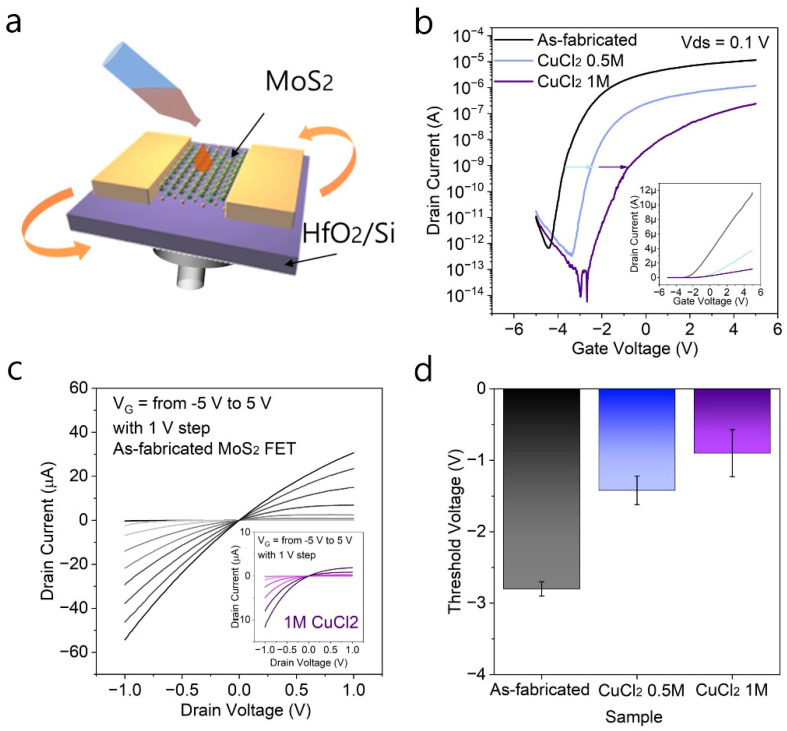
(**a**) Schematic description of CuCl_2_ doping on MoS_2_ FETs. (**b**) The transfer characteristics, (**c**) output characteristics, (**d**) threshold voltages of MoS_2_ FETs before and after CuCl_2_ doping.

To understand the effect of CuCl_2_ doping on the electrical properties of FETs based on a 2D MoS_2_ channel, the electrical properties were measured before and after doping the MoS_2_ FETs with CuCl_2_. The FET devices were fabricated on a HfO_2_/Si substrate using photolithography and metal deposition using a thermal evaporator. Figure 4a shows the schematic description of the doping process of MoS_2_ FETs. The as-fabricated MoS_2_ FETs were spin-coated with CuCl_2_ solution. Figure 4b shows the representative transfer curve, drain-source current, *I_DS_*, as a function of the gate voltage, *V_G_*, which is plotted on a logarithmic scale at the applied drain voltage of *V_DS_* = 0.1 V. The inset of Figure 4b shows the transfer curve in a linear scale. The as-fabricated MoS_2_ FET showed n-type transfer characteristics and a large ON/OFF ratio above 10^7^. From the transfer characteristics, we have estimated a field effect mobility using $\mu_{FE}=\frac{L}{WC_{ox}V_{ds}}\frac{dI_{ds}}{dV_{gs}}$, where *L* is channel length, *W* is channel width, and *C_ox_* is the gate capacitance of 309.9 nF cm^−^^2^. The field effect mobility was measured to be 12.3 cm^2^/Vs, which is in good agreement with reported values for a back-gated transistor using CVD-grown MoS_2_ monolayers. It can be observed from Figure 4b that the CuCl_2_-doped MoS_2_ FETs shows a gradual decrease in both *ON* and *OFF* current as the CuCl_2_ is doped onto the MoS_2_ FETs.

Such behavior was also found when an output curve, the drain-source current versus drain-source voltage on a linear scale, was measured as shown in Figure 4c (inset image shows the output curve of 1 M CuCl_2_ doped MoS_2_ FET). The output curve of as-fabricated MoS_2_ FET shows a high ON current and a linearly dependent drain current showing good Ohmic contact between MoS_2_ and electrodes. As the CuCl_2_ is doped onto MoS_2_ FETs, the channel conductance is largely decreased, showing a p-type doping effect of CuCl_2_ on MoS_2_, in accordance with transfer curve measured in Figure 4b.

Following the reduced ON current and channel conductance, it was also shown that as CuCl_2_ was doped onto MoS_2_ FETs, threshold voltage (*V_th_*) was shifted towards positive voltages from -2.85 V to -1.45 V and -0.9 V as 0.5 M and 1 M CuCl_2_ was doped onto MoS_2_ FETs as shown in Figure 4d. Using the changes in the V_th_, the change in carrier concentrations upon CuCl_2_ doping can be calculated using the parallel-plate capacitor model [3,9], $N_{doping}=\frac{C\left|\Delta Vth\right|}{e}$, where *C* is the gate capacitance, $\Delta V_{th}$ is the change in the *V_Th_* after the CuCl_2_ is doped onto the device compared to the as-fabricated MoS_2_ FET, and e is the elementary charge. The amount of doping concentration was estimated to be 2.72 × 10^12^ cm^−2^ and 3.77 × 10^12^ cm^−2^ when 0.5 M CuCl_2_ and 1 M CuCl_2_ were doped onto MoS_2_ FETs, respectively. The electrical analysis of MoS_2_ FETs before and after CuCl_2_ doping show that CuCl_2_ is an effective p-type dopant for MoS_2_ that can be easily employed using simple spin coating of different concentrations of CuCl_2_ solution.

## 4. Conclusions

To conclude, we have demonstrated simple and effective p-type doping on the 2D MoS_2_ FETs by simply spin coating the device with CuCl_2_ solution at ambient conditions. The effect of CuCl_2_ doping was confirmed analytically through Raman, PL, and XPS measurements. The p-type doping on the MoS_2_ channel showed largely decreased channel conductance and the shift in threshold voltages towards positive gate voltages in back-gated MoS_2_ transistors. It was also shown that the amount of doping can be simply controlled by the CuCl_2_ concentrations in a solution containing ethanol. The results and findings present an important pathway towards designing a CMOS circuit based on 2D FETs and other nanomaterials.

## Data Availability

Not applicable.
